# Anoikis-Associated Lung Cancer Metastasis: Mechanisms and Therapies

**DOI:** 10.3390/cancers14194791

**Published:** 2022-09-30

**Authors:** Jing Wang, Zhijie Luo, Lizhu Lin, Xinbing Sui, Lili Yu, Cong Xu, Ruonan Zhang, Ziming Zhao, Qianru Zhu, Bo An, Qiao Wang, Bi Chen, Elaine Lai-Han Leung, Qibiao Wu

**Affiliations:** 1 State Key Laboratory of Quality Research in Chinese Medicines, Faculty of Chinese Medicine, Macau University of Science and Technology, Macau 999078, China; 2 Northeast Asia Research Institute of Traditional Chinese Medicine, Changchun University of Chinese Medicine, Changchun 130117, China; 3 The First Clinical Medical College, The First Hospital Affiliated, Guangzhou University of Chinese Medicine, Guangzhou 510405, China; 4 School of Pharmacy, Department of Medical Oncology, Hangzhou Normal University, Hangzhou 311121, China; 5 Cancer Center, Faculty of Health Science, MOE Frontiers Science Center for Precision Oncology, University of Macau, Macau 999078, China; 6 Guangdong-Hong Kong-Macao Joint Laboratory for Contaminants Exposure and Health, Guangdong University of Technology, Guangzhou 510006, China; 7 Zhuhai MUST Science and Technology Research Institute, Zhuhai 519031, China

**Keywords:** lung cancer, anoikis, apoptosis, neoplasm metastasis, molecular mechanism therapy

## Abstract

**Simple Summary:**

Anoikis is a programmed cell death process resulting from the loss of interaction between cells and the extracellular matrix. Therefore, it is necessary to overcome anoikis when tumor cells acquire metastatic potential. In lung cancer, the composition of the extracellular matrix, cell adhesion-related membrane proteins, cytoskeletal regulators, and epithelial–mesenchymal transition are involved in the process of anoikis, and the initiation of apoptosis signals is a critical step in anoikis. Inversely, activation of growth signals counteracts anoikis. This review summarizes the regulators of lung cancer-related anoikis and explores potential drug applications targeting anoikis.

**Abstract:**

Tumor metastasis occurs in lung cancer, resulting in tumor progression and therapy failure. Anoikis is a mechanism of apoptosis that combats tumor metastasis; it inhibits the escape of tumor cells from the native extracellular matrix to other organs. Deciphering the regulators and mechanisms of anoikis in cancer metastasis is urgently needed to treat lung cancer. Several natural and synthetic products exhibit the pro-anoikis potential in lung cancer cells and in vivo models. These products include artonin E, imperatorin, oroxylin A, lupalbigenin, sulforaphane, renieramycin M, avicequinone B, and carbenoxolone. This review summarizes the current understanding of the molecular mechanisms of anoikis regulation and relevant regulators involved in lung cancer metastasis and discusses the therapeutic potential of targeting anoikis in the treatment of lung cancer metastasis.

## 1. Introduction

Lung cancer is one of the most malignant tumors worldwide, characterized by high morbidity and mortality [1]. The outcomes for patients with metastatic lung cancer are poor, with a 5-year survival rate of <5% [2]. Tumor metastasis is a complex multistep process, including migration from cancer tissue, intravasation, survival in the circulatory system, extravasation, homing, and metastatic colonization [3,4,5]. To finish the initial migration step, tumor cells must interrupt cell–cell and cell–stromal interactions, become flexible, and acquire plasticity to navigate the tumor stroma mechanically [3]. However, migrating tumor cells are then confronted with a survival challenge resulting from the loss of cell adhesion. Extracellular matrix (ECM) adhesion plays an essential role in cell differentiation, proliferation, and motility [6]. Normal cells can only grow and differentiate in the correct environment within the tissue, and they eliminate themselves by apoptosis in abnormal environments.

Anoikis (Greek for “homeless”) is apoptosis induced by loss of cell adhesion to the ECM or inappropriate cell adhesion [7]. Differentiated cells of multicellular organisms grow in appropriate tissue environments, and when cells are lost or leave their native environment, apoptosis signal transduction occurs. Anoikis occurs in malignancies, cardiovascular disease, diabetes, and infectious diseases; it includes metastatic detachment of tumor cells, cardiomyocyte detachment in heart failure, and endothelial cell detachment; other examples are hyperglycemia in diabetic microangiopathy and disruption of ECM-mediated cell loss of adhesion in infectious diseases [8,9,10]. Cancer cells escape tumor tissue and overcome anoikis to adapt to uncontrolled growth elsewhere in tumor metastasis. Tumor cells can require resistance to anoikis; this escape protects them in the lymphatic and circulatory systems [11,12]. Anoikis resistance is critical for tumor progression and metastasis, and tumor cells can escape from anoikis in several ways [13]. Therefore, we reviewed animal and cell models of anoikis in lung cancer and relevant transmembrane proteins and intracellular regulators. Then natural and synthetic products that promote anoikis were summarized.

## 2. Methodology

A literature search was performed in PubMed and Google scholar from January 1990 to September 2022. The search term was “anoikis”. A secondary search was conducted by screening the list of articles that met the inclusion criteria. The keywords were “lung cancer” OR “lung adenocarcinoma” OR “non-small-cell lung cancer” OR “small-cell lung cancer”. The obtained 362 articles were screened, and 79 duplicate articles, 23 review articles, and 113 irrelevant studies were removed. One hundred forty-seven relevant studies were included after reading the abstract. Among them, 101 were related to the mechanism of anoikis, and 46 were related to therapies for anoikis in lung cancer. Finally, we organized the tables, wrote the text, and created figures to summarize mechanisms and therapies of anoikis-associated lung cancer metastasis according to the SANRA [14].

## 3. Regulators and Mechanism of Anoikis in Lung Cancer

**Figure 1:**
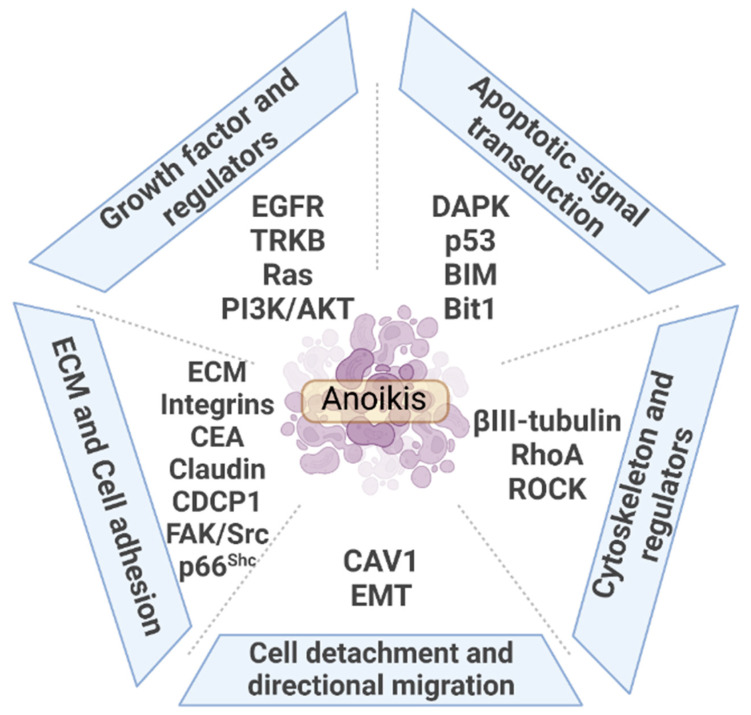
Overview of the regulation of anoikis in lung cancer metastasis.

The present work reviews the regulation of lung cancer on anoikis, including extrinsic and intrinsic regulators, which mainly includes five aspects provided in Figure 1. Anoikis originates from changes in the external environment triggered by cell detachment from the primary site. ECM components and cell adhesion-related membrane proteins act as sensors to transmit cell signals intracellularly. Regulatory proteins of cell adhesion and cytoskeleton, p66^shc^ and Ras homolog family A (RhoA), activate cells to initiate apoptosis. Activation of apoptosis signal transduction is an executor of anoikis. By contrast, proteins that increase cell adhesion, tight junction biological processes, and cytoskeletal proteins are influential factors of anoikis. Proliferation signals help tumor cells escape anoikis. The epithelial–mesenchymal transition (EMT) process causes loss of cell adhesion, accompanied by proteins such as caveolin 1 (CAV1) that promote cell directional migration and ultimately mediate anoikis resistance. The role of the above five aspects on anoikis in lung cancer metastasis is discussed in detail (Table 1).

### 3.1. ECM and Cell Adhesion

#### 3.1.1. ECM

**Figure 2:**
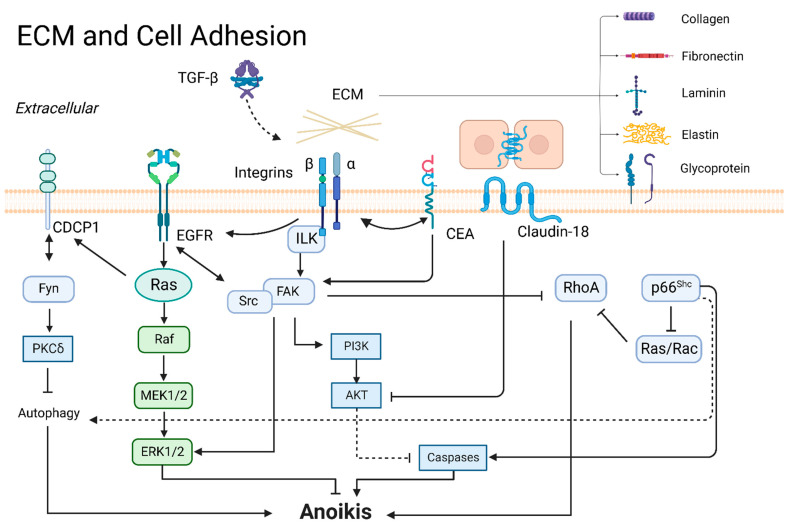
ECM and cell adhesion in anoikis.

ECM comprises fibronectin, laminins, collagens, elastin, and several other glycoproteins. They bind with cell adhesion receptors to form a three-dimensional macromolecular network, which regulates various cell functions, including survival, growth, morphology, and migration [15]. Tumor cells undergo anoikis due to loss or inappropriate cell adhesion to ECM [16]. Acquisition of anchorage-independent survival requires tumor cells survival when detached from the ECM matrix [17,18]. In different artificially mimicked ECM compositions, A549 cells exhibited different sensitivity to doxorubicin and anoikis in vitro. Downregulation of focal adhesion kinase (FAK) signaling is accompanied by anoikis sensitization [18]. As for ECM components, fibronectin is upregulated for cell aggregate formation during cell detachment, which enhances anoikis resistance in lung cancer. Fibronectin knockdown decreases glycoproteins, including desmoglein-2, desmocollin-2/3, and plakoglobin, to increase anoikis [19]. Laminin 5 expression is significantly correlated with advancement along the alveolar wall growth pattern and metastasis in lung adenocarcinoma. Laminin 5 activates integrin/FAK signaling to induce anoikis resistance in several lung adenocarcinoma cell lines suspended in soft agar-coated dishes [20]. Liu et al. showed that collagen XVII cooperated with laminin 5 to activate FAK and mediate suspension survival. The authors found that activation of PP2A/STAT3 promotes collagen XVII-induced suspension survival, which determined anoikis resistance and initial metastasis in A549 cells and malignant lung cancer pleural effusion mouse models [21]. Highly expressed collagen IV facilitated liver metastasis in lung cancer patients. Burnier et al. investigated lung-metastasizing M-27 cells and found that collagen IV silencing enhanced anoikis through the integrin α2/FAK axis in vitro and reduced liver metastasis by inoculation of tumor cells into the intrasplenic/portal system in vivo [22]. These findings suggest that upregulation of ECM components can mediate cell survival and re-adhesion to promote metastasis of tumor cells, primarily through integrin/FAK. A summary is presented in Figure 2.

#### 3.1.2. Integrins

Integrins are a family of cell surface heterodimeric receptors consisting of noncovalently linked α- and β-subunits that determine the receptor affinity for ECM. Integrin-mediated tumor stroma sensing, stiffening, and remodeling are critical steps in cancer progression that support tumor invasion, acquiring tumor stem cell properties, and drug resistance [23]. Integrins help metastatic cancer cells facilitate anchorage-independent survival and resist anoikis [24,25], and abnormal integrin expression contributes to several cancers’ metastasis [26]. In addition, integrin signaling was stimulated by growth factors to induce crosstalk. Integrin β3 was activated by transforming growth factor-β1 (TGF-β1) to increase the epidermal growth factor receptor (EGFR) tyrosine kinase inhibitor resistance, then EMT and anoikis resistance was observed in lung cancer cell lines [27]. Several studies have proven that FAK was a major downstream effector resisting anoikis through the Src or ERK pathway [20,22,25]. Furthermore, membrane proteins carcinoembryonic antigen (CEA) and cellular retinoic acid binding protein 2 (CRABP2) engage in integrin signaling to suppress anoikis [28]. Wu et al. found that CRABP2 promoted integrin β1/FAK/ERK signaling and inhibited anoikis. Overexpressed CRABP2 increased lymph node and liver metastasis through in vivo tail vein injection of lung cancer cell models [25].

#### 3.1.3. CEA

CEA family members are involved in the biological function of intercellular adhesion [29]. First, Camacho-Leal et al. found that the expression of CEA is closely related to integrins, and deleting the CEA functional domain weakened the cross-linking of integrin α5β1 and cell adhesion to fibronectin, which led to anoikis [28]. Subsequently, antibody-mediated CEA deletion attenuated specific binding to integrin α5β1. The co-clustered CEA and integrin α5β1 activated integrin-linked kinase (ILK), AKT, and ERK/mitogen-activated protein kinase (MAPK) pathways to impair anoikis [30]. Furthermore, CEA reduces anoikis through the downregulation of the intrinsic cell death pathway, and the inactivation of caspase-9 and -3 [31]. The above studies demonstrate the effect of CEA on anoikis resistance. In addition, CEA-related cell adhesion molecule 6 (CEACAM6) is upregulated in lung adenocarcinoma patients with poor outcomes. Homophilic interactions of CEACAM6 between lung cancer cells and the tumor microenvironment could inhibit anoikis through Src/FAK pathway activation [32].

#### 3.1.4. Claudins and Occludin

Claudins and occludin are the essential components of the tight junctions, which establish the paracellular barrier. Serglycin interacted with CD44-enhanced claudin-1 expression to promote vimentin expression, EMT, and anoikis resistance [33]. Conversely, claudin-18 promoted cell death and anoikis by inhibiting phosphorylated 3-phosphoinositide-dependent protein kinase-1 (PDK1) and pAKT levels in several lung cancer cell lines [34]. Occludin is an integral membrane linker protein. The thyroid transcription factor 1 (TTF-1) enhances the promoter of occludin and mediated anoikis [35].

#### 3.1.5. CUB-Domain-Containing Protein 1 (CDCP1)

CDCP1 is an Src family kinase-binding phosphoprotein implicated in promoting metastasis via anoikis inhibition [36,37]. Phosphorylation of CDCP1 enhanced anchorage-independent growth of A549, PC14, H520, and H322 in a colony formation assay on soft agar. A549 cells with knockdown CDCP1 injected into the tail veins of a mouse model were found to have fewer metastatic nodules [36]. Regarding the mechanism of CDCP1 against anoikis, a few studies have proven that Ras activated CDCP1; subsequently, Tyr734 of CDCP1 bound to Fyn and was phosphorylated to activate protein kinase Cδ (PKCδ) and inhibit autophagy, helping tumor cells to escape apoptosis [36,38,39].

#### 3.1.6. FAK

FAK is a ubiquitously expressed non-receptor tyrosine kinase that regulates cellular functions from embryonic development to wound healing, cell adhesion, cell migration, and angiogenesis [40,41]. For cell adhesion, FAK is recruited by integrins that form a dual kinase complex. The complex responds to signals from ECM components, including fibronectin and collagen [20,22,25]. FAK modulates both cell death- and survival-related pathways to resist anoikis. On the one hand, FAK binds to Src or directly activates the phosphatidylinositide-3-kinase (PI3K)/AKT, ERK/MAPK, and p38/MAPK pathways for survival during cell detachment [22,25,42]. On the other hand, FAK upregulates the phosphorylation of p190RhoGAP and inhibits RhoA-triggered pro-apoptotic phosphorylation of BCL-2-interacting mediator of cell death (BIM) to escape from anoikis [10]. Olfactomedin III is highly expressed in anoikis-resistant lung cancer cell lines and promotes the phosphorylation of FAK to keep procaspase-3 from activation [43]. Conversely, apoptosis signals inhibit FAK. X-linked ectodermal dysplasia receptor (XEDAR) was an essential effector downstream of p53 and negatively regulated FAK to induce anoikis [44].

#### 3.1.7. Tyrosine-Protein Kinase Src

Cellular functions of Src kinases are involved in the cell cycle, proliferation, differentiation, adhesion, and angiogenesis [45]. These functions are mediated by Src kinases acting as membrane adhesion molecular switches, linking various extracellular signals to intracellular signaling pathways [46]. Src phosphorylation creates anoikis resistance and causes lung cancer cells to “float” in lymph nodes [47]. Src is a membrane-attached molecule regulated by several pathways to attenuate anoikis. FAK recruits Src to inhibit anoikis as previously mentioned [42]. It is also reported that Pyk2 appeared to be the critical downstream effector of Src and induced metastasis in lung cells [48]. Furthermore, Wei et al. found that Src Tyr418 phosphorylation triggered phosphorylation of p130^Cas^ and caused anchorage-independent growth and metastasis in lung cancer [49]. 

Some regulators counteract anoikis through Src, including zinc finger protein 2 (ZIC2) and growth factor receptors. ZIC2 promoted tumorigenesis and anoikis resistance of non-small-cell lung cancer (NSCLC) by FAK/Src signaling, verified in NSCLC patients [50]. Platelet-derived growth factor receptor (PDGFR) increased the phosphorylation of Src Tyr419 and induced metastasis in lung cells [48]. In addition, EGFR mediated phosphorylation of c-Src to attenuate anoikis [51]. Family with sequence similarity 188 member B (FAM188B, a deubiquitinase) prevented degradation in phospho-EGFR, phospho-Src, and phospho-ERK to inhibit anoikis in lung cancer cells [52].

#### 3.1.8. p66^Shc^

p66^Shc^ is an SHC adapter protein 1 (SHC1) gene-encoding protein, localized to focal adhesions to permit anchorage-dependent growth and promote anoikis function as a tumor metastasis suppressor [53]. Expression of p66^Shc^ in lung cancer samples correlated with good outcomes and anoikis [54]. p66^Shc^ promotes anoikis in lung cancer through the perception of cell attachment, inhibition of proliferation, and promotion of apoptosis. p66^Shc^ restrains Ras and Rac1 hyperactivation and increases RhoA activation to require focal adhesion and restore anoikis [55,56]. Typically, p66^Shc^ did not affect cell death in adherent cells. During cell detachment, p66^Shc^ translocated into mitochondria and bound to cytochrome c to release and cause death [55]. On the other hand, in the absence of adhesion conditions, p66^Shc^ induced autophagy and activated apoptosis-associated protein cleavage of caspase-7 and poly (ADP-ribose) polymerase (PARP) by inhibiting phosphorylation of ERK1/2 [57,58]. DNA methylation in promoter-mediated epigenetic repression of p66^Shc^ increased cancer cell survival and tumor progression [59]. Aiolos is an Ikaros zinc finger family member associated with epigenetic modifications [60]. Aiolos acts as an oncogene to disrupt enhancer–promoter interactions of p66^Shc^ to inhibit transcription [61]. Aiolos also silences the zinc finger transcription factor PR domain containing 1 (PRDM1), resulting in anoikis resistance [62]. Similarly, zinc-finger E-box binding protein 1 (ZEB1) repressed the p66^Shc^ promoter. Furthermore, ZEB1 potentiates EMT and blocks anoikis by increasing vimentin and decreasing E-cadherin and β-catenin [63].

### 3.2. Growth Factors and Regulators

#### 3.2.1. EGFR

EGFR is a transmembrane protein that transduces intracellular growth factor signals, promotes tumor proliferation and metastasis, and is an essential target of current lung cancer therapy. In the absence of cell adhesion, normal cells lose expression of EGFR and induce apoptosis; by contrast, tumor cells do not exhibit loss of EGFR during detachment [64]. In growth signaling, EGFR acquires survival and anoikis resistance by activating Ras, ERK, and PI3K/AKT pathways [51,65,66]. EGFR has crosstalk through cell-adhering proteins or inhibits apoptosis signaling to inhibit anoikis. EGFR and integrins cooperatively inhibited anoikis by downregulating the BIM during cell detachment from the ECM [27,64]. NADPH oxidase 4 (NOX4) is a reactive oxygen species (ROS)-generating enzyme. NOX4 increased the activation of EGFR through ROS generation; a soft agar colony assay showed that si-NOX4 and si-EGFR attenuated Src expression and enhanced anoikis [51]. Regulators that mediate EGFR protein degradation are also involved in the regulation of anoikis. E3-ubiquitin ligase c-Cbl is an essential ligand for EGFR degradation. CCN family protein 2 (CCN2) bound EGFR and recruited E3-ubiquitin ligase for EGFR ubiquitination and degradation [66]. FAM188B prevented EGFR from degrading to cause lung cancer cells to re-adhere to the ECM [52].

#### 3.2.2. Neurotrophic Tyrosine Kinase Receptor 2 (NTRK2/TrkB)

TrkB is a neurotrophin that serves as a growth factor regulating embryonic stem cells and promoting tumorigenesis in cancer cells. TrkB is upregulated in anoikis-resistant lung-derived tumor cells isolated from malignant ascites [67]. TrkB activates EMT, tumorigenesis, and lung metastasis and attenuates anoikis via the twist/snail axis in breast and lung cancer [68]. The expression of TrkB and E-cadherin had an inverse relationship in a panel of lung adenocarcinoma samples [69]. Cancer-derived TrkB mutations partly altered the functional characterization of the protein. TrkB^T695I^ and TrkB^D751N^ in colon cancer-derived mutants showed less activity than wild-type TrkB, and the function of TrkB^L138F^ in lung cancer and TrkB^P507L^ in breast cancer was indistinguishable from wild-type [70].

#### 3.2.3. Ras

Ras is a small GTPase that includes HRas, KRas, and NRas; these are the most common oncogenes in human cancer [71]. Raf is a downstream effector of Ras, mediating proliferation signaling, including phosphorylation of MEK1/MEK2 and MAPK/ERK1/2 [72]. p66^Shc^ and RhoB restrained Ras hyperactivation to enhance anoikis as mentioned before [56,65,73]. Ras-mutated NSCLC cell lines displayed high CDCP1 expression connected with anoikis resistance. The Ras/ERK/CDCP1 axis represses anoikis in lung cancer [38]. Furthermore, the HRasV12- or KRasV12-induced lung tumorigenesis and anti-anoikis effect required the expression of cisplatin resistance-related protein-9 (CRR9) [74].

#### 3.2.4. PI3K/AKT

**Figure 3:**
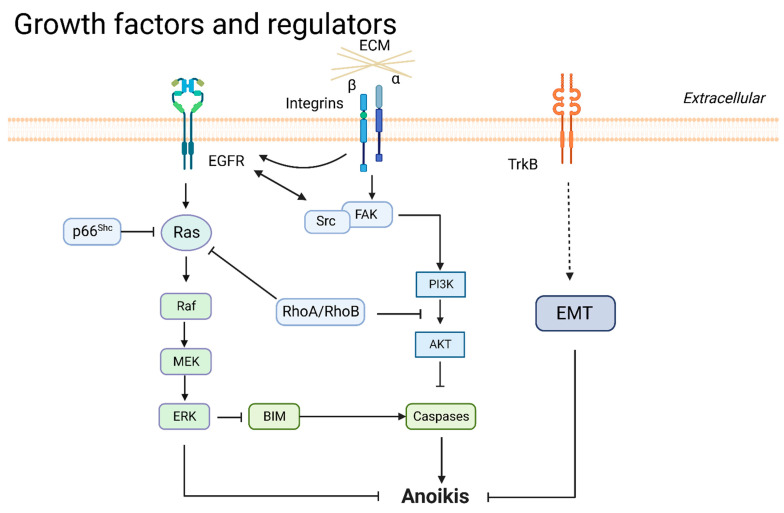
Growth factors and regulators in anoikis.

The activation of the PI3K/AKT pathway can cause lung cancer cells’ anoikis resistance [75]. Numerous proteins regulate anoikis resistance through the PI3K/AKT pathway. CEA, TrkB, FAK/Src, and βIII-tubulin promote phosphorylation of AKT for anoikis resistance as mentioned. Then, the activation of PI3K/AKT promotes EMT and inhibits caspases, allowing cells to escape anoikis [30,31,42,70,76,77]. Furthermore, farnesylated AKT1 suppresses anoikis and causes resistance to DNA-reactive agents [78]. Some regulators inhibit anoikis through the PI3K/AKT pathway. Interactions between PI3K and CRR9 activated AKT and BCL-XL accumulation, inducing anoikis resistance [74]. Contactin 1 enhanced the PI3K/AKT pathway, promoting EMT and anoikis resistance [77]. IGF-I-mediated IGF-IR phosphorylation activated downstream AKT to play an anti-apoptotic role [79]. IL-13 receptor subunit alpha-2 (IL13Rα2) activated PI3K/AKT and transcriptional coactivator with PDZ-binding motif, resulting in anoikis resistance. The overexpression of IL13Rα2 was associated with poor outcomes in lung cancer [80]. Noncoding RNA participates in PI3K/AKT regulation. lncRNA VAL binding to vimentin activates AKT, inducing lung cancer anchorage-independent growth and metastasis. lncRNA VAL prevents vimentin from undergoing Trim16 E3 ligase-mediated degradation [81]. A summary of growth factors and regulators is presented in Figure 3.

### 3.3. Cytoskeleton and Regulators

#### 3.3.1. βIII-Tubulin

βIII-tubulin is a microtubule protein constituent of the cytoskeleton. It contributes to tumor metastasis and chemotherapy resistance [82]. βIII-tubulin contributed to anti-anoikis and metastasis in lung cancer. High levels of βIII-tubulin inhibited the phosphatase and tensin homolog deleted on chromosome ten (PTEN) and enhanced phosphorylation of AKT to induce tumor spheroid outgrowth and anoikis resistance in NSCLC cells [76].

#### 3.3.2. Rho and Rho-Associated Kinase (ROCK)

**Figure 4:**
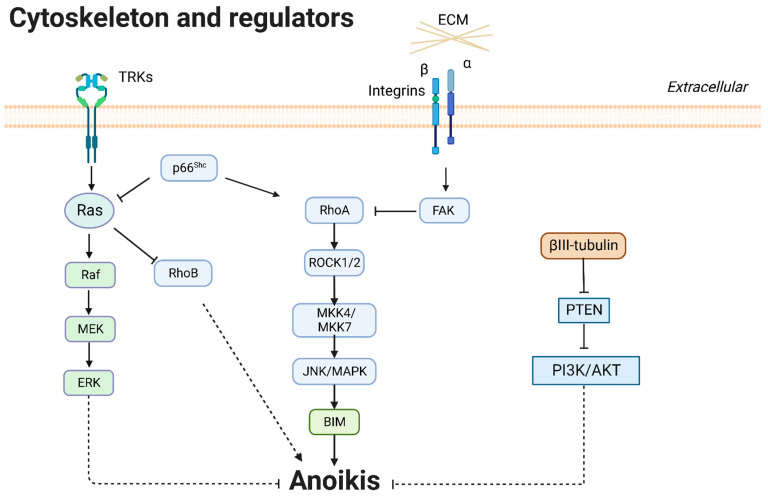
Cytoskeleton and regulators in anoikis.

The Rho family of GTPases are small signaling G proteins from the Ras superfamily. RhoA regulates cell morphology change, cell–matrix adhesion, and cytoskeletal reorganization [83,84]. Cell adhesion molecules regulate RhoA; activation of FAK counteracted RhoA inducing anoikis [10], and p66^Shc^ promoted RhoA, causing anoikis [56]. RhoB can inhibit invasion and proliferation and induce anoikis, whereas growth signals Ras, ERK, and PI3K/AKT counteract the effects of RhoB [65,73]. In summary, RhoA and RhoB promote anoikis and antagonize Ras. ROCK kinases are critical downstream effectors of Rho GTPases [85]. Haun et al. showed that RhoA/ROCK activated MKK4/MKK7 and JNK to promote BIM phosphorylation and caspase-3 processing, activating the anoikis signaling pathway [10]. A summary of cytoskeleton and regulators is presented in Figure 4.

### 3.4. Cell Detachment and Directional Migration

#### 3.4.1. CAV1

CAV1 is a vital component of plasma membrane caveolae; it undergoes extracellular changes and regulates caveola-dependent signaling and endocytosis [86]. CAV1 is a cellular membrane raft structure that responds to external environmental stimuli such as ROS, shear stress, and mechanical stress. In addition, CAV1 can regulate cell polarization and directional migration [87]. CAV1 functions as a membrane adaptor to kinase Fyn in integrin signaling, critical for anchorage-dependent cell growth [88]. Several studies have proven that CAV1 overexpression enhanced anchorage-independent growth in H460 cells in vitro [89,90]. Myeloid leukemia-1 (MCL-1) is an anti-apoptotic protein required to escape anoikis in several tumors, including breast cancer, osteosarcoma, and melanoma [91,92]. CAV1 interacted with MCL-1 to prevent degradation and resist anoikis [93].

Tumor cells suffer fluid shear stress through the venous and lymphatic systems, which can acquire anoikis resistance. CAV1 can help cells overcome fluid shear stress and anoikis [94,95]. Free radicals stimulate CAV1 to a large extent during cell detachment. Oxidative stress products, including nitric oxide (NO) and hydrogen peroxide (H_2_O_2_), inhibited CAV1 degradation to resist anoikis [90,96]. NO-mediated S-nitrosylation of CAV1 is a protein modification that stabilizes CAV1 protein and protects it from ubiquitination [89]. As feedback, anoikis resistance enhanced CAV1 expression [97,98]. H_2_O_2_ inhibited the formation of the CAV1–ubiquitin complex through the consumption of catalase and N-acetylcysteine to cause anoikis resistance in H460 cells [96,99,100,101].

The relationships between miRNAs and CAV1 participate in anoikis. CAV1 mRNA is directly inhibited by miR-1827; downregulated CAV1 induces anoikis in A549 cells [102]. CAV1 and lipid raft-dependent endocytosis promote the transfer of miR-222-3p. Exosomes drive miR-222-3p targeting the promoter of suppressor of cytokine signaling 3 (SOCS3) to enhance gemcitabine resistance, migration, and anti-anoikis effects in vivo and in vitro [103].

#### 3.4.2. EMT

EMT is characterized by epithelial cells undergoing remarkable morphologic changes to the mesenchymal phenotype, leading to increased motility and invasion in cancer [104]. Loss of the epithelial marker E-cadherin makes cells lose attachment and antagonizes the response to anoikis after cell detachment [105,106]. Some features of EMT, including loss of the epithelial cell–cell junction, reorganization of the actin cytoskeleton, and gain of directional migration capability, are critical for anoikis resistance [107,108]. Upregulation of N-cadherin, vimentin, twist, snail, and ZEB1, feature proteins of EMT, inhibits anoikis [63,68,109]. In summary, EMT is a process of adaptation to anchorage-independent growth, which is a factor against anoikis.

Some critical proteins (i.e., integrin, claudin-1, p66^Shc^, RhoA, CAV1, contactin 1, and TrkB) are involved in EMT in the context of anoikis, as previously mentioned [63] [27,33,68]. RhoA downstream signaling can affect actin remodeling and prevent the formation of contractile stress fibers during EMT [109]. Several pathways (i.e., the PI3K/AKT, Notch, and Wnt pathways) are related to EMT. Contactin 1 promotes EMT and anoikis resistance by enhancing the PI3K/AKT pathway [106]. Notch-1 enhanced the expression of vimentin and snail to suppress anoikis [110]. Fas apoptotic inhibitory molecule 2 (FAIM2) induced EMT and anoikis resistance through the Wnt/β-catenin pathway in NSCLC bone metastasis [111]. NO exposure upregulated EMT and enhanced anoikis resistance, as shown by anoikis assays with poly-HEMA-coated plates [90].

### 3.5. Apoptotic Signal Transduction

#### 3.5.1. Death-Associated Protein Kinase (DAPK)

DAPK is a Ser/Thr protein kinase that mediates apoptosis signaling with upregulation of interferon-γ, tumor necrosis factor-α, and Fas to initiate apoptosis and anoikis [112,113]. CCN2 assisted DAPK in overcoming the inhibition by MAPK/ERK signaling [52].

#### 3.5.2. p53

As a principal apoptosis regulator of tumors, p53 plays a pivotal role in anoikis-mediated cell death. p53-dependent anoikis has been demonstrated in several cell lines in lung cancer [44,114]. p53 upregulated cleavage caspase-3 to inhibit integrin α6β4, AKT signaling, and tumor metastasis [115]. XEDAR is an effector downstream of p53. p53 binds to intron 1 of the XEDAR gene and promotes transcription [44]. The expression of p53 and liver kinase B1 (LKB1) is positive feedback; however, loss of p53 or p14 cooperating with mutant Kras results in the inactivation of LKB1 [116,117,118]. Regulation of the p53 pathway is essential for tumor treatment, and some drugs interfere with the p53 pathway, as detailed below.

#### 3.5.3. BIM

The pro-apoptotic protein BIM, a BH3-only protein, is a critical executor of apoptosis in anoikis. BIM disrupts the outer mitochondrial membrane to induce apoptosis, a response to anchorage-independent growth [119]. 14-3-3ζ knockdown in A549 cells was accompanied by upregulation of the pro-apoptotic protein BIM rather than Bad and susceptibility to anoikis. BIM inhibits BCL-2, Bcl-xL, and MCL-1, leading to Bax activation and anoikis [120]. In addition, when RhoA/ROCK senses cell detachment, signals are transmitted to BIM to initiate the apoptosis process [10].

#### 3.5.4. Bit1

**Figure 5:**
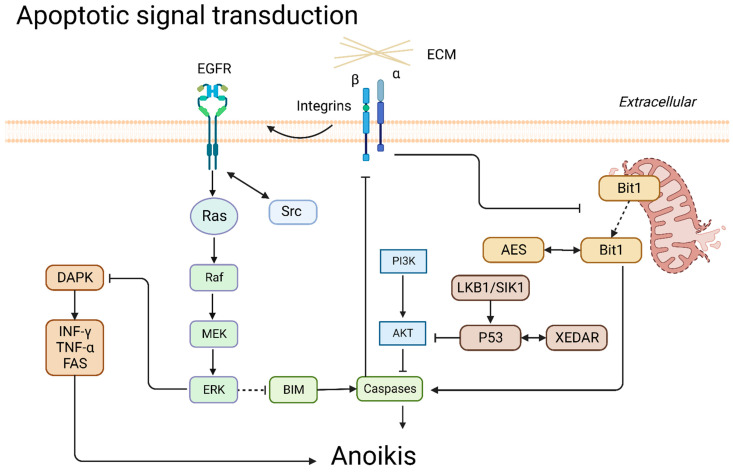
Apoptotic signal transduction in anoikis.

BCL-2 inhibitor of transcription 1 (Bit1) is named for the ability to reduce BCL-2 promoter activity. After integrin- or ECM-mediated cell attachment, Bit1 is released from the mitochondria to the cytoplasm and forms a complex with the transcriptional regulator amino-terminal enhancer split (AES) protein to initiate cell death against anchorage-independent growth [121,122]. In addition, the Bit1/AES complex upregulated E-cadherin to inhibit EMT and enhanced anoikis in lung cancer cells in vitro [121,123]. Transducin-like enhancer of split 1 (TLE1) sequestrates AES into the nucleus to reduce the formation of the Bit1/AES complex. Bit1 induced cytoplasmic translocation and degradation of TLE1 during anoikis [122,124,125]. A summary is presented in Figure 5.

### 3.6. Other Regulators

#### 3.6.1. LKB1

Several studies demonstrated that LKB1 accumulation promotes anoikis in tumors [126]. LKB1 decreases anchorage-independent growth of A549 and HeLa cells, suggesting that LKB1 is a tumor suppressor and anti-metastasis factor [127]. Salt-inducible kinase 1 (SIK1) is an LKB1-dependent kinase that inversely correlates with poor prognosis and distal metastases in breast cancer. The SIK1/LKB1 complex promotes p53-dependent anoikis and suppresses metastasis in lung cancer [114]. Meanwhile, loss of LKB1 is connected to poor prognosis in lung cancer. In LKB1-deficient lung cancer, pleomorphic adenoma gene 1 (PLAG1) mediates the upregulation of a glutaminolysis enzyme, glutamate dehydrogenase 1 (GDH1), and calcium/calmodulin-dependent protein kinase kinase 2 bound to AMP-activated protein kinase (AMPK). PLAG1/GDH1-mediated activation of AMPK confers anti-anoikis effects by inhibiting the mechanistic target of rapamycin (mTOR) [128,129,130].

#### 3.6.2. Other Regulators

There are several studies on the negative regulation of anoikis. Phosphorylated Pyk2, MET amplification, glycolysis regulator (TIGAR), and BUB1B are anoikis suppressors; overexpression of these factors attenuated anoikis in lung cancer [131,132,133,134]. PARP1 binds near the promoter region of CLDN7 and S100A4 to upregulate expression at the transcriptional level, which induces DNA repair and attenuates anoikis [135]. Activating transcription factor 4 binds to the heme oxygenase 1 promoter and coordinates with nuclear factor erythroid 2-related factor 2 to induce cytoprotective autophagy, ROS suppression, and anoikis resistance [136]. Deletion of NAD(P)H/quinone oxidoreductase 1 (NQO1) increases ROS formation and anoikis sensitization in NSCLC. NQO1 potentiates glycometabolism, enhancing cell proliferation and anoikis resistance [137,138]. ΔNp63α, the major isoform of p63, protects tumor cells from oxidative stress, DNA damage, anoikis, and ferroptosis through transcriptional upregulation of glutathione metabolism genes *GCLC*, *GSS*, *IDH2*, and *GPX2* [139]. Interleukin enhancer-binding factor 2 (ILF2) directly binds to the PTEN upstream regulatory region, promoting anchorage independence in NSCLC [140]. Activated transcription factor Spi-B (SPIB) enhances SNAP47 transcription and mitigates anoikis in lung cancer [141]. Keratin 14 contributes to anoikis repression via upregulation of gastrokine 1 in lung cancer [142]. Loss of CoA desaturase (SCD1) induces cellular damage and anoikis in circulating tumor cells (CTCs) [143]. Macrophage-stimulating protein (MSP) binds to recepteur d’origine nantais (RON) to promote liver metastases by affecting the organ microenvironment in SCLC [144].

As for promoting anoikis factors, Krüppel-like factor 12 (KLF12) promotes cell cycle transition through the S phase to induce anoikis [145]. Trim62 E3 ligase is an anoikis protective factor; the loss of Trim62 E3 ligase promotes EMT, tumorigenesis, and anti-anoikis effects in lung cancer [146]. Src homology 2-b3 (SH2B3) is reduced in lung cancer tissues and cells. SH2B3 interacts with Janus kinase 2 (JAK2) and Src homology region 2-containing protein tyrosine phosphatase 2 to inhibit JAK2/STAT3 and PI3K/AKT signaling pathway-mediated anoikis in lung cancer [147].

**Table 1:** Molecular factors and mechanisms related to anoikis in lung cancer.

Factors	Anoikis	Model(s)	Mechanism	Ref
Fibronectin	↓	A549, H460, and H1975	Fibronectin upregulated cell desmosomal interactions.	[19]
Laminin 5	↓	A549, PC-14, LC-2/ad, RERF-LC-KJ, NCI-H322, and NCI-H358	Laminin 5/integrin/FAK signaling pathway activated the expression of EGFR.	[20]
Collagen XVII	↓	A549	S727 phosphorylation of STAT3 activated collagen XVII to maintain the stability of laminin 5.	[21]
Collagen IV	↓	Lung-metastasizing M-27 cells	Collagen IV activated integrin α2/FAK and increased reactivity to IGF-I.	[22]
CRABP2	↓	L1, C9F6, and H1650	CRABP2 coupled with HuR promoted integrin β1/FAK/ERK signaling.	[25]
Integrin β3	↓	HCC827, H1975, A549, H292, and H1299	The activation of the TGFβ1/integrin β3 axis overcame acquired resistance to EGFR-TKIs and anoikis.	[27]
CEA	↓	L6 and LR-73	CEA inactivated caspase-9 and caspase-8 and enhanced PI3K/AKT pathway.	[28,30,31]
Claudin-1, Serglycin, and CD44	↓	H1299, H322, H358, H23, H928, H460, and A549	Serglycin interacting with CD44 enhanced claudin-1 expression to promote EMT and anoikis resistance.	[33]
Claudin-18	↑	A549, RERF-LC-AI, IA-LM, WA-hT, PC-3, and RERF-LC–MS	Claudin-18 inactivated PDK1 and phospho-AKT levels.	[34]
Occludin and TTF-1	↑	H441, A549, H1299	TTF-1 transactivated occludin to promote anoikis.	[35]
CDCP1	↓	A549, PC14, H322, H520, and H157	Tyr734-phosphorylated CDCP1 regulated PKCδ and inhibited autophagy.	[36,38,39]
FAK	↓	A549	Phosphorylated tyrosine sites (Tyr397, Tyr861, Tyr925) in FAK bound to intracellular proteins of Src and stimulated PI3K/AKT pathway, MAPK/ERK pathway, and MAPK/p38 pathway.	[42]
Olfactomedin III	↓	Poorly differentiated human squamous carcinoma, named DLKP cell line	Olfactomedin III upregulated phospho-FAK and phospho-Paxillin and kept procaspase-3 from activation.	[43]
Src	↓	SK-LU-1, H522, H1437, A549, H460, and H1792	EGFR, PDGFR, and ZIC2 increased the phosphorylation of Src to induce anoikis resistance.	[48,50,52]
p130^Cas^	↓	A549 and H1792	Src contributed to the phosphorylation of p130^Cas^ in the tumor cells.	[49]
ZIC2	↓	HFL1, Calu-3, H1975, H1395, H520, A549, H1299, H226, SK-MES-1, and BEAS-2B	ZIC2 promoted tumorigenesis and anoikis resistance of NSCLC by Src/FAK signaling.	[50]
FAM188B	↓	A549, H1299, H1975	FAM188B potentiated the stabilization of EGFR expression.	[52]
p66^Shc^	↑	LLC, H69, and H209	p66^Shc^ potentiated autophagy through cleavage of caspase 7 and PARP.	[57,58]
Aiolos	↓	A549, H1155	Aiolos disrupted the enhancer of p66^Shc^ at the transcription level.	[61]
PRDM1	↑	HUVEC, A549, Beas-2B, H209, H69, H1155	PRDM1 was silenced by aiolos.	[62]
EGFR	↓	A549, H1703, Calu-6, H460, H358, HCC2279, BEAS-2B	EGFR mediated phosphorylation of c-Src and ERK to attenuate anoikis.	[51,52,66]
NOX4	↓	A549, H1703, Calu-6, H460, H358, HCC2279, BEAS-2B	NOX4 increased the activation of Src and EGFR, attenuating anoikis	[51]
TrkB	↓	Primary cell cultures derived from malignant pleural effusions, L1 sarcoma cells from a primary, spontaneous lung tumor in Balb/c	TrkB inhibited EMT. The expression levels of TrkB and E-cadherin were opposite in lung adenocarcinoma samples.	[68,69,70]
Ras	↓	A549, PC14, H322, H520, and H157	Ras/ERK signaling activated CDCP1 to induce anoikis resistance. Ras/ERK pathway was regulated through CAV1.	[38,88]
βIII-tubulin	↓	H460 and A549	High levels of βIII-tubulin enhanced phospho-AKT activity via PTEN suppression.	[76].
RhoA	↑	BEAS-2B, LLC, H69, and H209	RhoA counteracted FAK and was activated by p66^Shc^, causing anoikis.	[10,56]
RhoB	↑	NIH-3T3, A549	RhoB was suppressed by Ras/PI3K/AKT. Suppression of RhoB resulted in anoikis resistance.	[65,73]
ROCK	↑	BEAS-2B	RhoA/ROCK activated MKK4/MKK7/JNK/BIM to promote apoptosis.	[10]
NO	↓	H460, H23, H292	NO exposure enhanced EMT and upregulated CAV1, allowing escape from anoikis and promoting migration.	[89,90]
MCL-1	↓	NIH3T3, H460	Mcl-1 degradation activated anoikis. CAV1 interacted with MCL-1 to prevent MCL-1 from degradation via ubiquitination.	[92,93]
H_2_O_2_	↓	H460, G361	H_2_O_2_ inhibited the formation of the CAV1–ubiquitin complex.	[96,100]
miR-222-3p	↓	A549-GR	miR-222-3p directly targeted the promoter of SOCS3 to enhance gemcitabine resistance and anti-anoikis.	[103]
CAV1	↓	H460	CAV1, as a membrane adaptor to kinase Fyn, activated integrin signaling. CAV1 was negatively regulated by oxidative stress.	[88,90,96]
ZEB1	↓	HBECs, HepG2, H358, H1155, H1299, and A549	ZEB1 was a negative regulator of p66^Shc^.	[63]
Notch-1	↓	PC-9	Notch-1 enhanced EMT markers, including vimentin and snail.	[110]
FAIM2	↓	HARA, HARA-B4, H1395, A549, and NIH3T3	FAIM2 enhanced Wnt/β-catenin signaling pathway to facilitate EMT and anoikis resistance.	[111]
DAPK	↑	H460, A427, A549, and CL1-0	CCN2 promoted DAPK kinase activity and then activated the p53 pathway.	[52]
p53 and XEDAR	↑	A549, H1299, and HeLa	p53 enhanced cleavage caspase 3 and AKT activity and promoted XEDAR expression to inhibit integrin/FAK signaling.	[44,115]
CRR9	↓	A549, H838	CRR9 interacted with PI3K to activate oncogene Ras.	[74]
Contactin 1	↓	H446, H526	Contactin 1 activated the AKT pathway to promote EMT phenotype.	[77]
MMP-7	↓	BALB/c 3T3	MMP-7 degraded IGFBP-3 and activated the AKT pathway, resulting in anoikis.	[79]
IL13Rα2	↓	HBE-135, HBE-154, PC9, H1975, A549, HTB-57, H2170, H1299, H358, H3255, H1838, and HCC827	IL13Rα2 activated PI3K/AKT and TAZ, resulting in migration, invasion, and anoikis resistance.	[80]
lncRNA VAL	↓	A549, HCC827, H1650, H596, H1975, H1299, H292, H2009, and H2030	lncRNA VAL bound to vimentin and inhibited vimentin degradation.	[81]
Farnesylated AKT1	↓	MCF10A and A549	Farnesylated AKT1 suppressed anoikis and caused resistance to DNA-reactive agents, but not altered cell cycle (M-phase) specific chemotherapeutics.	[78]
BIM	↑	NIH3T3, A549, BEAS-2B	BIM induced endangerment of mitochondria and apoptosis.	[92,119]
14-3-3ζ	↓	A549	Deficiency of 14-3-3ζ upregulated BAD and BIM and decreased MCL-1 to increase BAX, cleaved caspase 7, and cleaved caspase 3 toward tumor cell anoikis.	[120]
Bit1	↑	A549	Bit1/AES complex initiated cell death complex and inhibited EMT through upregulation of E-cadherin.	[121,122]
TLE1	↓	A549	TLE1 interfered with AES formation Bit1/AES complex.	[122,124]
NQO1	↓	A549, H292	NQO1 decreased ROS formation and anoikis sensitization.	[137,138]
ΔNp63α	↓	HCC95	ΔNp63α protected cells from oxidative stress, DNA damage, anoikis, and ferroptosis through upregulation of glutathione metabolism.	[139]
ILF2	↓	HUVEC-C, HBEC-5i, BEAS-2B, A549, H460, H1155, and H1299	ILF2 directly inhibited PTEN via binding to its upstream regulatory region.	[140]
SPIB	↓	A549, H1703, H1975, H446, H520, H226, SK-MES-1, H460, H1299	Activated SPIB directly enhanced SNAP47 transcription in lung cancer cells and increased anoikis resistance.	[141]
Keratin 14	↓	Kras^G12D^/Trp53^L/L^ cell lines from de novo KP tumors	Gastrokine 1 cooperated with keratin 14, inhibited anoikis, and promoted cancer metastasis.	[142]
SCD1	↓	H460	Loss of SCD1 induced cellular damage and anoikis.	[143]
MSP	↓	SBC-5, H1048	MSP phosphorylated RON to promote liver metastases in lung cancer.	[144]
KLF12	↑	A549, H23, H460	KLF12 promotes cell cycle transition through the S phase to induce anoikis.	[145]
TRIM62	↑	Trim62^+/−^ and Trim62^−/−^ C57BL/6J mice	The loss of TRIM62 synergizes with K-Ras mutation, promoting EMT, tumorigenesis, and metastasis in lung cancer.	[146]
SH2B3	↑	A549, NCI-H358, NCI-H1650, NCI-H460, NCI-H1688, Calu-1; A549 and NCI-H1688 tumor-bearing nude mouse	SH2B3 inhibits JAK2/STAT3 and PI3K/AKT signaling pathways to induce anoikis in lung cancer.	[147]

↓: downregulation of anoikis; ↑: upregulation of anoikis.

## 4. Therapy for Anoikis in Lung Cancer

**Figure 6:**
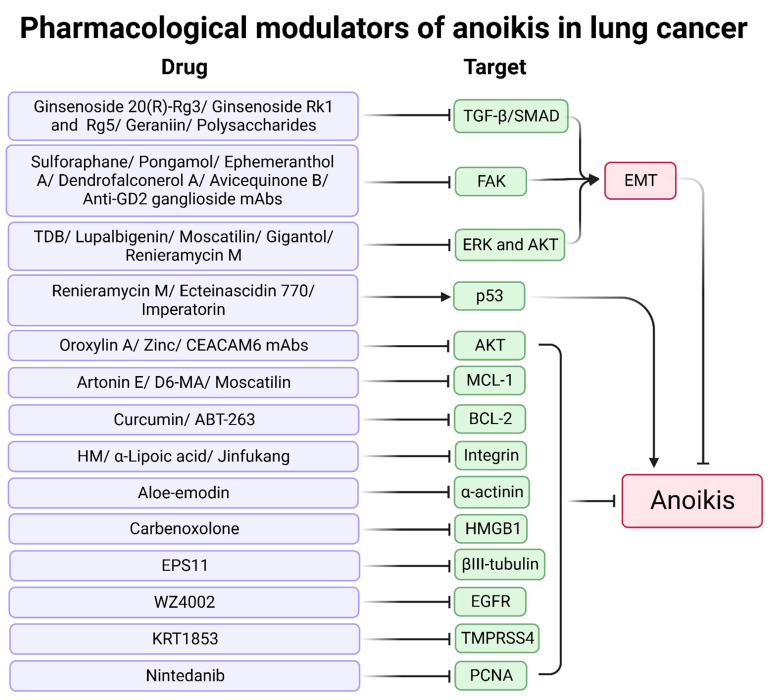
Pharmacological modulators of anoikis in lung cancer.

Investigations in lung cancer models demonstrate the potent anti-tumor activity of treatments such as monoclonal antibodies (mAbs), natural products, chemosynthetic drugs, and multi-component drugs against lung cancer metastasis via activation of anoikis (Table 2 and Figure 6).

### 4.1. Natural Products

Numerous studies suggest that drugs enhancing anoikis are derived from natural products, of which small molecule compounds are the most common, including natural herbs such as Thai medicine, Chinese medicine, and marine natural products. The primary targets of natural products for anoikis include inhibition of MCL-1, AKT, FAK, and EMT.

For MCL-1 and anoikis-related pathways, renieramycin M and ecteinascidin 770, isolated from Thai tunicate *Ecteinascidia thurstoni*, exhibit promising anticancer activity via a p53-dependent pathway and inhibition of MCL-1 and BCL-2 [148,149]. Imperatorin from *Angelica dahurica* root was first reported by Pithi et al.; it enhances protein expression of p53 and Bax and reduces MCL-1, which promotes anoikis and cell death sensitization in several lung cancer cell lines [150]. Artonin E is extracted from the bark of *Artocarpus gomezianus*; it enhances the anoikis of lung cancer cells in a dose-dependent manner by downregulating MCL-1 [151].

Inhibition of AKT is an effective anoikis promoter. Several natural products inhibit lung cancer metastasis through the PI3K/AKT pathway. Oroxylin A, a flavonoid isolated from *Scutellaria* root, induces anoikis via the inhibition of the c-Src/AKT pathway and then increases the loss of mitochondrial membrane potential [152]. 4,5,4′-Trihydroxy-3,3′-dimethoxybibenzyl (TDB), isolated from *D. ellipsophyllum*, inhibits the anchorage-independent growth of lung cancer cells. TDB inhibits EMT through the inactivation of phospho-ERK and phospho-AKT [153]. Lupalbigenin, isolated from *Derris scandens*, inhibits phospho-AKT, phospho-ERK, and BCL-2 for sensitivity to anoikis in lung cancer [154].

FAK is associated with cell adhesion, and inhibition of FAK contributes to anoikis. Sulforaphane is an isothiocyanate found in cruciferous vegetables that promotes anoikis and cell death. Sulforaphane reduces FAK, AKT, and β-catenin and upregulates p21 to induce anoikis in lung cancer cells with wild-type p53 [155]. Pongamol is a chalcone derived from the *T. purpurea* stem extract that mitigates EMT and permits anoikis through downregulation of the FAK and AKT/mTOR signaling pathways [156]. EPS11 is a purified polysaccharide from a crude extract of marine bacterial polysaccharide; it inhibits the expression of βIII-tubulin at the transcription level, triggering suppression of the downstream effectors phospho-PKB and phospho-AKT in vitro and in vivo [157].

EMT promotes the progression and metastasis of lung cancer, and some natural products target anoikis by inhibiting EMT. Ginsenoside Rg3 promotes anoikis and EMT inhibition via the TGF-β/SMAD pathway in A549 [158]. Ginsenosides Rk1 and Rg5 suppress EMT through downregulation of MMP2/9 activity, inhibitory actions of Smad2/3, and the NF-kB/ERK pathway in A549 cells [159]. Jorunnamycin A is a bis-tetrahydroisoquinoline quinone isolated from the blue sponge *Xestospongia* sp.; it reduces EMT and anchorage-independent survival by regulating apoptosis-related proteins and EMT markers. p53 and E-cadherin are upregulated after treatment with Jorunnamycin A [160].

The active compounds from *Dendrobii caulis* inhibit lung cancer through anoikis. Moscatilin, gigantol, and ephemeranthol A exhibit strong anti-EMT and anti-migration activity involving suppression of ERK, AKT, and CAV1 [161,162,163]. Dendrofalconerol A, extracted from *Dendrobium falconeri* (Orchidaceae), sensitizes anoikis-induced cell death involving inhibitory effects of phospho-FAK and Rho-GTP in H460 cells [164]. Four bibenzyls isolated from the stem of *Dendrobium pulchellum* (chrysotobibenzyl, chrysotoxine, crepidatin, and moscatilin) facilitate anoikis and inhibit metastasis in lung cancer cells [165]. Phenolic compounds from *Dendrobium ellipsophyllum* (4,4′-dihydroxy-3,5-dimethoxybibenzyl, 4,5,4′-trihydroxy-3,3′-dimethoxybibenzyl, chrysoeriol, and luteolin) have cytotoxic and anoikis-related activities [166].

There are also natural products used for anti-tumor effects that stimulate anoikis. Aloe-emodin induces anoikis by interfering with the downregulation of α-actinin and the MAPK pathway (i.e., JNK, ERK1, and p38) and upregulation of PKCδ [167,168]. Curcumin increases BCL-2 ubiquitination and degradation, sensitizing cells to detachment-induced anoikis [169]. Geraniin, extracted from *Phyllanthus amarus*, exhibits anti-anoikis activity via TGF-β suppression [170].

Crude extracts of plants and Chinese medicine formulas target anoikis in lung cancer. A polysaccharide extract obtained from rough extraction of persimmon leaves inhibited EMT and anoikis resistance through the canonical TGF-β/SMAD pathway and inhibited the MAPK pathway [171]. Oat avenanthramides inhibited EGFR, suppressing lung cancer cell growth and migration [172]. Jinfukang is a traditional Chinese medicine prescription for lung cancer treatment; it inhibits the integrin/Src pathway, suppressing ECM–receptor interaction and focal adhesion-related genes [173]. Modified Bu-Fei decoction inhibits hypoxia-inducible factor 1α (HIF-1α) signaling and angiogenin-like protein 4 (ANGPTL4) to inhibit A549 cells anoikis and lung metastasis of LLC-bearing mice [174].

### 4.2. Synthetic Products

Chemosynthetic products have more precise targets and lower active concentrations for treating malignant tumors; these include the inhibitors of some targets such as BCL-2 and EGFR. BCL-2 inhibitor ABT-263 acts as adjunctive therapy to promote cell death-related proteins and anoikis [175]. WZ4002, a third-generation EGFR inhibitor, causes anoikis and inhibits lung cancer metastasis [176]. TMPRSS4 serine protease inhibitor KRT1853 promotes anoikis of lung cancer cells by inhibiting the JNK/MAPK, PI3K/AKT, and NF-κB pathways [177]. Nintedanib is available for idiopathic pulmonary fibrosis treatment. High-dose nintedanib (5 uM) promotes anoikis and apoptosis via downregulation of PCNA in NSCLC [178].

Derivatives of natural products were studied in lung cancer treatment, and modified natural products had anti-tumor activity. Renieramycin M exhibits anoikis activity by inhibiting phospho-ERK, phospho-AKT, BCL-2, and MCL-1 [179]. Avicequinone B (chemically synthesized from lawsone) inhibits MCL-1 and BCL-2 through the integrin/FAK/Src axis to promote anoikis [180]. Carbenoxolone (chemically derived from glycyrrhizin) enhances anoikis by inhibiting high mobility group box 1 (HMGB1) [181]. α-l-Rhamnose monosaccharide derivative (D6-MA), a novel synthetic derivative of digitoxin, has anticancer activity in lung cancer cell lines; it attenuated MCL-1 expression via glycogen synthase kinase-3β-mediated ubiquitin proteasomal degradation [182].

Chemical synthesis products and chemical element supplements also show good anti-anoikis activity. N,N-Bis(5-ethyl-2-hydroxy benzyl) methylamine (HM) enhances anoikis by targeting the integrin β3/FAK/AKT axis [183]. α-Lipoic acid decreases integrin β1 and β3 to induce anoikis [184]. Zinc sensitizes to anoikis by inhibiting AKT and CAV1 [185].

Macromolecules related to biological agents treat anoikis in lung cancer. Several mAbs inhibit lung cancer metastasis. Anti-ganglioside (GD2) mAbs reduce FAK phosphorylation levels and promote p38/MAPK for SCLC treatment [186]. LN-332 mAbs reduce anoikis resistance and migration, associated with tumor cell–matrix interaction [187]. CEACAM6 mAbs decrease phospho-AKT to promote anoikis in lung cancer. CEACAM6 mAbs with paclitaxel treatment markedly decreased tumor growth by 40% to 80% compared to CEACAM6 mAbs alone and were more effective in CEACAM6-targeting albumin-based nanoparticles [188,189].

**Table 2:** Pharmacological modulators of anoikis in lung cancer.

Modulators	Tested Model(s)	Mechanism	Ref
Renieramycin M	H460	p53 activation	[148]
Ecteinascidin 770	H23, H460	p53 activation	[149]
Imperatorin	H23, H292, and A549	p53 activation and MCL-1 downregulation	[150]
Artonin E	H460, A549, and H292	MCL-1 downregulation	[151]
Oroxylin A	A549	Inhibitory effect of c-Src/AKT pathway	[152]
TDB	H292	EMT inhibition via inactivation of phospho-ERK and phospho-AKT	[153]
Lupalbigenin	H460	Phospho-AKT, phospho-ERK, and BCL-2 inhibition	[154]
Sulforaphane	A549 and CL1-5	Reduction of FAK, AKT, and β-catenin and upregulation of p21	[155]
Pongamol	H460	Inhibition of EMT through FAK and AKT/mTOR signaling pathways	[156]
EPS11	H460, A549, and H1299 A549 xenograft with BALB/c-nu mice	Inhibitory effect on βIII-tubulin	[157]
Ginsenoside 20(R)-Rg3	A549	EMT inhibition through the TGF-β/SMAD pathway	[158]
Ginsenosides Rk1 and Rg5	A549	Inhibition of TGF-β1-induced EMT	[159]
Jorunnamycin A	H460, H292, and H23	Suppression of EMT and apoptosis-related protein	[160]
Moscatilin	H460	EMT inhibition involves repression of ERK, AKT, MCL-1, and CAV1	[161]
Gigantol	H460	EMT inhibition involves suppression of ERK, AKT, and CAV1	[162]
Ephemeranthol A	H460	EMT inhibition via FAK/AKT pathway	[163]
Dendrofalconerol A	H460	Suppression of phospho-FAK and Rho-GTP	[164]
Chrysotobibenzyl, chrysotoxine, crepidatin, and moscatilin	H23	Anti-anoikis activities	[165]
4,4′-Dihydroxy-3,5-dimethoxybibenzyl, 4,5,4′-trihydroxy-3,3′-dimethoxybibenzyl, chrysoeriol, and luteolin	H292	Anti-anoikis activities	[166]
Aloe-emodin	H460	Downregulation of α-actinin and MAPK pathway (JNK, ERK1, p38) and upregulation of PKCδ	[167,168]
Curcumin	H460	Upregulation of the degradation of BCL-2	[169]
Geraniin	A549	EMT inhibition via TGF-β1	[170]
Polysaccharide	A549	Inhibition of TGF-β1-induced EMT	[171]
Oat avenanthramides	A549	Suppression of EGFR	[172]
Jinfukang	H1975	Suppression of integrin/Src pathway	[173]
Modified Bu-Fei decoction	A549 and LLC-bearing mice	Inhibition of ANGPTL4 expression through suppressing HIF-1α signaling	[174]
ABT-263	LC-KJ, HCC827, H1650, and H1975	A BCL-2 inhibitor enhanced Src inhibitors	[175]
WZ4002	HCC827 and H1975	A third-generation EGFR inhibitor	[176]
KRT1853	H322	TMPRSS4 serine protease inhibitors, repression of JNK/MAPK, PI3K/AKT, and NF-κB pathways	[177]
Nintedanib	A549, H1299, and H460	Downregulation of PCNA	[178]
Renieramycin M	H460	Downregulation of phospho-ERK, phospho-AKT, BCL-2, and MCL-1	[179]
Avicequinone B	H460, H292, and H23	Diminution of integrin/FAK/Src, MCL-1, and BCL-2	[180]
Carbenoxolone	C57BL/6J mice injected with LLC	Inhibition of HMGB1	[181]
D6-MA	H460	MCL-1 downregulation	[182]
HM	H292	Inhibition of integrin β3	[183]
α-Lipoic acid	H460	Integrin β1 and β3 inhibition	[184]
Zinc	H460	Downregulation of AKT and CAV1	[185]
Anti-GD2 ganglioside mAbs	NCI-417, ACC-LC-171, and ACC-LC-96 in vitro	FAK reduction and p38 activation	[186]
Anti-LN-332 mAbs	KLN-205 tumors in DBA/2 mice	Blocking cell–matrix interaction	[187]
CEACAM6 mAbs	Balb/C xenotransplanted A549	Inhibitory effects on phospho-AKT and upregulation of paclitaxel chemosensitivity	[188,189]

## 5. Discussion

**Figure 7:**
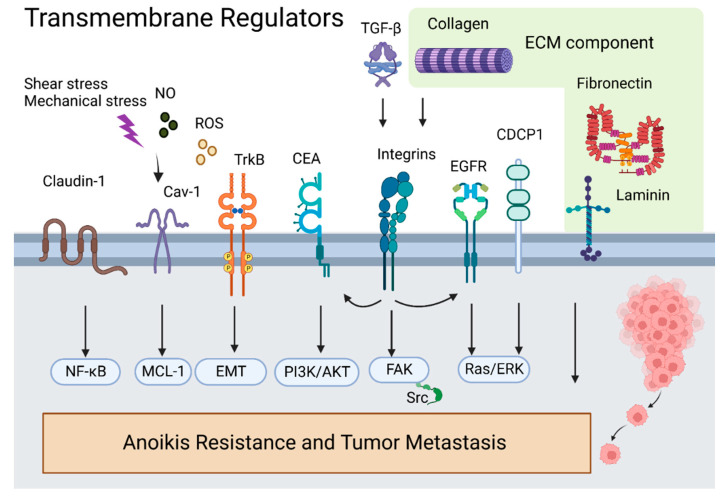
Overview of transmembrane regulators in anoikis-associated lung cancer metastasis.

Anoikis is a critical biological process that antagonizes lung cancer metastasis. Tumor metastasis begins when cells detach from their native environment and adapt to new sites through blood vessels, lymphatics, or body cavities. Anoikis prevents tumor cells from detachment and re-adhesion to new matrices in incorrect locations or the new organism. During this process, the ECM, including collagen IV; laminin 5; fibronectin; and cell membrane proteins including integrins, CEA, EGFR, CDCP1, and CAV1, acts as a sensor and is the first site to receive cell detachment signals. CEA and EGFR engage in crosstalk with integrins and promote metastasis. Integrins can trigger FAK/Src, an essential pathway for inhibiting anoikis. CAV1 is a membrane adapter to kinase Fyn in integrin signaling; it interacts with MCL-1 to inhibit anoikis (Figure 7).

**Figure 8:**
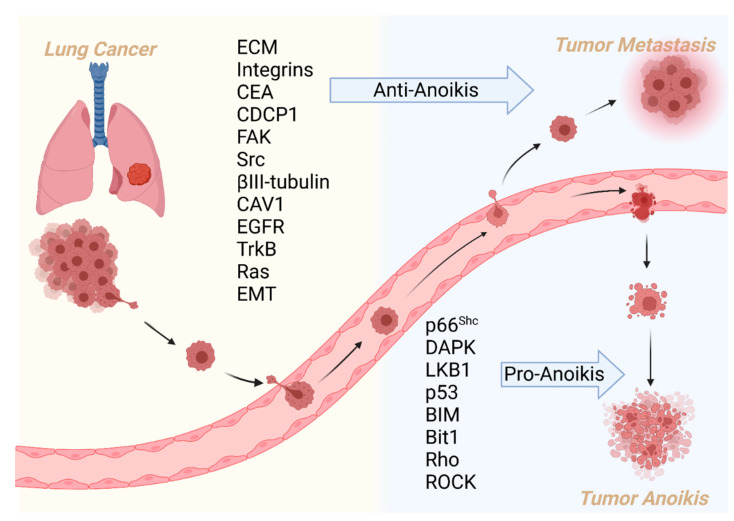
Overview of primary regulators in anoikis-associated lung cancer metastasis.

p66^shc^ is a focal adhesion regulatory protein that initiates apoptosis signals for anoikis. Apoptosis-related proteins (i.e., BIM, P53, DAPK, and caspases) are critical targets driving anoikis. Inhibitors of apoptosis proteins MCL-1, Bit, and BCL-2 suppress anoikis. CEA, TrkB, FAK/Src, and βIII-tubulin inhibit anoikis through upregulation of the PI3K/AKT pathway. Next, growth factors negatively regulate anoikis, and TGF-β activates integrins to trigger intracellular apoptosis resistance. EFGR and TrkB activate ERK/MAPK or Ras/Raf/ERK signaling pathways to counteract anoikis. Furthermore, cytoskeleton regulator RhoA and the downstream effector ROCK antagonize Ras and FAK signaling and promote apoptotic signaling (Figure 8).

Because of evidence of anoikis reducing metastasis, preclinical investigations in lung cancer focus on targeting anoikis using novel small molecule compounds (natural and synthetic products), mAbs, and repurposed FDA-approved compounds. Therapeutic targets include increased p53 and inhibition of TGF-β/SMAD, FAK, AKT, and MCL-1. Despite several preclinical studies on anoikis-related inhibitors, a gap urgently needs to be filled, including an analysis of compounds that combat lung cancer metastases in vivo.

## 6. Conclusions

Anoikis plays an important role in lung cancer metastasis and is associated with tumor progression and therapy failure. The composition of the extracellular matrix, cell adhesion-related membrane proteins, cytoskeletal regulators, and epithelial–mesenchymal transition are involved in the process of anoikis, and the initiation of apoptosis signals is a critical step in anoikis. Several natural and synthetic products, including artonin E, imperatorin, oroxylin A, lupalbigenin, sulforaphane, renieramycin M, avicequinone B, and carbenoxolone, exhibit pro-anoikis potential. This review provides an overview of the major regulators and mechanisms of anoikis in lung cancer and discusses the therapeutic potential of targeting anoikis in the treatment of lung cancer.
